# Analog Resistive Switching Phenomena in Titanium Oxide Thin-Film Memristive Devices

**DOI:** 10.3390/ma18153454

**Published:** 2025-07-23

**Authors:** Karimul Islam, Rezwana Sultana, Robert Mroczyński

**Affiliations:** Warsaw University of Technology, Institute of Microelectronics and Optoelectronics, Koszykowa 75, 00-662 Warsaw, Poland; rezwana.sultana@pw.edu.pl (R.S.); robert.mroczynski@pw.edu.pl (R.M.)

**Keywords:** titanium oxide thin film, reactive magnetron sputtering, analog resistive switching, non-volatile, memristor

## Abstract

Memristors with resistive switching capabilities are vital for information storage and brain-inspired computing, making them a key focus in current research. This study demonstrates non-volatile analog resistive switching behavior in Al/TiO_x_/TiN/Si(n^++^)/Al memristive devices. Analog resistive switching offers gradual, controllable conductance changes, which are essential for mimicking brain-like synaptic behavior, unlike digital/abrupt switching. The amorphous titanium oxide (TiO_x_) active layer was deposited using the pulsed-DC reactive magnetron sputtering technique. The impact of increasing the oxide thickness on the electrical performance of the memristors was investigated. Electrical characterizations revealed stable, forming-free analog resistive switching, achieving endurance beyond 300 DC cycles. The charge conduction mechanisms underlying the current–voltage (I–V) characteristics are analyzed in detail, revealing the presence of ohmic behavior, Schottky emission, and space-charge-limited conduction (SCLC). Experimental results indicate that increasing the TiO_x_ film thickness from 31 to 44 nm leads to a notable change in the current conduction mechanism. The results confirm that the memristors have good stability (>1500 s) and are capable of exhibiting excellent long-term potentiation (LTP) and long-term depression (LTD) properties. The analog switching driven by oxygen vacancy-induced barrier modulation in the TiO_x_/TiN interface is explained in detail, supported by a proposed model. The remarkable switching characteristics exhibited by the TiO_x_-based memristive devices make them highly suitable for artificial synapse applications in neuromorphic computing systems.

## 1. Introduction

Brain-inspired computational systems based on artificial synapse networks have garnered significant attention due to their potential to surpass the traditional von Neumann computing paradigm. Memristive devices, as the core of artificial synapse networks, play a crucial role in mimicking the biological structure and function of synapses due to their non-volatility, precise and repeatable analog switching, and the potential for large-scale integration through crossbar array architectures [1,2,3]. Transition metal oxide-based memristive devices with resistive switching behavior in metal–insulator–metal (MIM) structures have been widely studied due to their scalability, low power consumption, CMOS compatibility, and simplicity [4]. In recent years, considerable efforts have been made to modulate the memristive properties or phase-transition dynamics of the active switching layer through photonic stimulation [5]. The resistance state of the active layer in a memristive device can be dynamically modulated by applying an external electrical bias [6]. Resistive switching (RS) primarily has two types: filamentary and homogeneous. Filamentary RS, also known as digital switching, involves the development of conductive filaments (CFs) within the active layer, with the switching process occurring between the filament tip and the electrode. This mechanism is widely documented and is notable for its abrupt SET/RESET transitions, distinct resistance states, and high resistance ratios, making it well suited for digital logic and data storage applications [7,8]. In contrast, homogeneous RS, also referred to as analog switching, exhibits a gradual variation in the conductance of the active layer. This behavior is attributed to a uniform current distribution over the whole cross-sectional area, indicating the absence of filament formation [9,10]. Such gradual modulation closely resembles synaptic behavior in biological systems, making it a promising approach for neuromorphic computing technologies [11,12]. These features are crucial for the crossbar-based hardware acceleration of neural networks, as discussed in several recent review articles that address device-level mechanisms, integration challenges, and system-level performance bottlenecks in neuromorphic architectures [13,14,15,16]. RS behavior has been reported in several metal oxide-based materials, including HfO_x_ [17], NiO_x_ [18], VO_x_ [19], TaO_x_ [20], TiO_x_ [21], and WO_x_ [22]. Among transition metal oxides, titanium oxide (TiO_x_) stands out due to its simple chemical structure, multiple switching modes, and diverse oxidation states. Due to a well-balanced combination of physical and chemical properties, along with excellent environmental compatibility, TiO_2_, a high-k dielectric with a wide bandgap and inherent oxygen vacancies, is particularly favorable for RS applications [23,24,25]. When oxygen atoms are removed from the TiO_2_ matrix, the remaining electrons occupy titanium’s conduction band, creating an oxygen-deficient structure with n-type conductivity [26]. Moreover, titanium-based perovskite oxides, such as lead zirconate titanate (PZT) films, have been extensively studied for high-frequency transducer applications [27]. Strontium titanate (SrTiO_3_) has gained attention as a promising material for RS devices [28]. TiO_2_ and TiO_2_-based thin films have been prepared using various techniques, including the sol–gel process [29,30], hydrothermal method [31], magnetron sputtering [32,33], electrochemical anodization [34], atomic layer deposition (ALD) [35], and several other fabrication approaches [36,37,38]. Magnetron sputtering (DC or RF) stands out as a relatively cost-effective deposition technique, offering precise control over film stoichiometry and uniformity in thickness across the substrate. Sputtering offers significant flexibility, enabling the deposition of a wide range of materials, including metals, semiconductors, and dielectric materials [39,40]. Despite ongoing research, several aspects of TiO_2_-based resistive random-access memory (RRAM) remain uncertain. Notably, the electrical performance of these devices often exhibits variability, and the underlying mechanisms governing resistive switching are not yet fully understood [41]. Interestingly, digital and analog resistive switching behaviors have been observed within the same devices, though the mechanism behind this is still unclear [42,43,44]. However, challenges related to device reliability and reproducibility persist. Therefore, improving the device’s performance and gaining deeper insight into its switching behavior remain essential research goals. While many researchers have recently reported that bilayer or doped TiO_x_ structures are effective to enhance the RS performance and improve the distribution of switching parameters [45,46], TiO_2_ continues to be a widely studied resistive switching material, with different deposition methods such as sputtering and sol–gel still actively explored [32,43,47]. This study intentionally employs a pure TiO_x_ layer to focus on its intrinsic switching behavior. This approach simplifies the material system, establishing a clear baseline for future investigations involving more-complex architectures. In this work, the TiO_x_ layer was deposited using pulsed-DC sputtering at room temperature to fabricate the Al/TiO_x_/TiN MIM structure, which is compatible with CMOS integration due to its low thermal budget and use of CMOS-friendly materials. To the best of our knowledge, this specific technique has not been extensively reported for TiO_x_-based resistive switching devices. In this article, the authors present the existence of relatively stable analog RS in TiO_x_ thin films deposited by pulsed-DC reactive sputtering at room temperature with Al and TiN electrodes at the top (TE) and bottom (BE), respectively. The electrical performance of fabricated Al/TiO_x_/TiN devices was investigated by varying the thickness of the TiO_x_ thin film. Comprehensive structural characterization of the TiO_x_ layer was conducted, and the current conduction mechanisms were analyzed based on the obtained electrical data. Moreover, a detailed discussion of the resistive switching mechanism was provided, supported by a proposed model.

## 2. Materials and Methods

**Figure 1:**
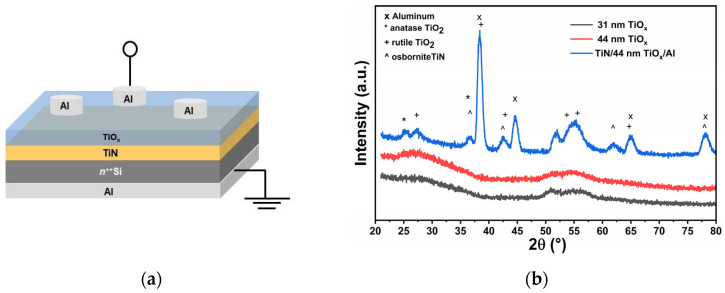
(**a**) Schematic diagram of Al/TiO_x_/TiN/Si(n^++^)/Al MIM device structure. (**b**) GI-XRD patterns of the TiO_x_ thin films deposited on Si(100) for different thicknesses and the TiN/TiO_x_ (44 nm)/Al tri-layer structure.

In this work, the Al/TiO_x_/TiN/Si(n^++^)/Al MIM devices were fabricated using a multi-step pulsed-DC magnetron sputtering technique. The substrate was a highly doped n-type silicon (Si) wafer with arsenic doping and a 0.004–0.008 Ω·cm resistivity. Initially, the silicon substrates underwent standard RCA cleaning, followed by a 1% hydrofluoric acid (HF) etching step for 1 min before being transferred into the load-lock chamber. A 60 nm titanium nitride (TiN) bottom electrode was first deposited on the Si substrates by pulsed-DC reactive magnetron sputtering from a metallic titanium target in an argon/nitrogen (Ar/N_2_) gas environment. The deposition was carried out at a chamber pressure of 3 mTorr using a 1:2 Ar:N_2_ gas flow ratio, and the sputtering power was set to 1000 W. Subsequently, without breaking the vacuum, a TiO_x_ active layer was deposited on top of the TiN layer. This was achieved by reactive sputtering of a metallic Ti target in an argon/oxygen (Ar/O_2_) atmosphere. A 3:2 Ar:O_2_ gas ratio was maintained to form a near-stoichiometric TiO_2_ film at a working pressure of 5 mTorr, again using a sputtering power of 1000 W. TiO_x_ thin films with thicknesses of 31 nm and 44 nm were obtained by conducting the deposition process for 7 and 10 min, respectively. All the depositions were carried out at room temperature. Specialized measurements were undertaken to elucidate the chemical composition and structural characteristics of the thin films. Film thicknesses were determined via spectroscopic ellipsometry using a Jobin-Yvon UVISEL (Horiba, Lille, France) system. The structure and crystallinity of the deposited films were characterized using grazing incidence X-ray diffraction (GI-XRD) with a PANalytical Empyrean Series 2 diffractometer equipped with a PIXcel3D detector (Panalytical, Almelo, The Netherlands), utilizing Cu-Kα radiation (λ = 1.54 Å). Measurements were conducted in the 2θ range of 20–80°, with a fixed angle of incidence. The elemental composition and the surface morphology were investigated through energy-dispersive X-ray spectroscopy (EDX) and field emission scanning electron microscopy (FE-SEM), utilizing a Hitachi SU-70 scanning electron microscope (Hitachi High-Technologies Corporation, Tokyo, Japan). To perform electrical characterization of the TiO_x_ films with thicknesses of 31 and 44 nm, top 100 nm thick Al contact pads were patterned using standard photolithography with ultraviolet (UV) exposure at 380 nm, followed by a lift-off process. The Al was deposited by magnetron sputtering using a high-purity (99.99%) aluminum target. This step was conducted at a power of 350 W with an argon gas flow rate of 25 sccm and a working pressure of 2 mTorr. To ensure improved electrical contact, the backside of the Si substrates was also coated with an aluminum layer. Electrical characterization was performed using a Keithley 4200 semiconductor parameter analyzer (Tektronix, Beaverton, OR, USA) equipped with a SUSS PM-8 probe station (SUSS MicroTec Semiconductor, Garching, Germany). The results presented in this work pertain to MIM devices featuring a top electrode diameter of 150 μm. The scheme of the Al/TiO_x_/TiN/Si(n^++^)/Al MIM device structure is presented in Figure 1a.

## 3. Results and Discussion

Figure 1b displays the GI-XRD patterns of the deposited thin films. The TiO_x_ films with thicknesses of 31 and 44 nm exhibited amorphous characteristics, as evidenced by the presence of broad halos centered around 26° and 55° in their diffraction patterns. In contrast, the GI-XRD pattern of the Al/TiO_x_ (44 nm)/TiN stack revealed distinct crystalline peaks corresponding to both rutile and anatase phases of TiO_2_, along with peaks from the Al top electrode and the TiN (osbornite) bottom electrode.

Specifically, the Bragg peak positions and corresponding Miller indices for the Al/TiO_x_/TiN tri-layer identified with the HighScore Plus suite (Database PDF-5+2025) include TiO_2_ anatase: 25.30° (011), 38.56° (112) (ref. code: 01-083-5914), TiO_2_ rutile: 27.08° (110), 38.66° (020), 43.45° (120), 53.77° (121), 55.83° (220), 64.75° (221) (ref. code: 98-016-5921), Al metal (TE): 38.48° (111), 44.72° (002), 65.10° (022), 78.24° (113) (ref. code: 98-024-0129), and TiN osbornite (BE): 36.73° (111), 42.67° (002), 61.93° (022), 78.13° (222) (ref. code: 04-018-2321). It was fascinating to see that the 44 nm TiO_x_ film displayed distinct crystallization when inserted between the TiN and Al electrodes, while it remained amorphous without electrodes. This crystallization is likely facilitated by metal-induced crystallization (MIC) or interfacial effects, where the neighboring metal layers may promote structural ordering through mechanisms such as localized thermal effects during deposition, stress-driven phase transformation, or diffusion-enhanced crystallization at the interfaces [48]. The insertion of the TiO_x_ layer between the TiN and Al electrodes resulted in stress formation within the TiO_x_ film. The nature of this stress was identified using the Δd parameter, estimated by the following equation [49]:

(1) $$\Delta d\;=\;\left(\frac{d-d^{\prime }}{d^{\prime }}\right)\times 100\%$$

where *d* represents the measured interplanar spacing, and *d*′ is the standard interplanar spacing value. A negative Δ*d* indicates compressive stress, while a positive value suggests tensile stress present in the thin film [49]. Based on X-ray diffraction (XRD) analysis, a Δ*d* value of −0.49% was observed, confirming the presence of compressive stress within the TiO_x_ layer.

**Figure 2:**
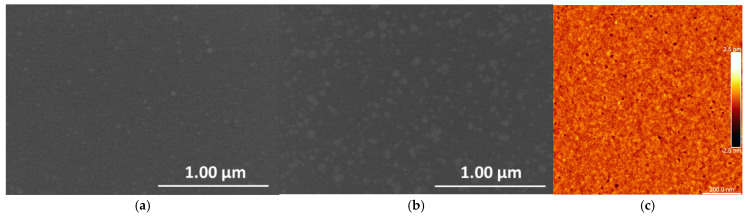
FE-SEM images of TiO_x_ thin films for two different thicknesses, (**a**) 31 nm and (**b**) 44 nm. (**c**) AFM topographic image of the 44 nm TiO_x_ thin film.

Figure 2a,b display FE-SEM images of TiO_x_ thin films with 31 nm and 44 nm thicknesses, respectively. Both films exhibit a uniform surface structure. The surface of the 31 nm TiO_x_ film appears very smooth, while the 44 nm film shows a comparatively rougher surface, attributed to a higher concentration of agglomerates [50]. Given that the 44 nm TiO_x_ film exhibits a rougher surface than the 31 nm film, only the 44 nm film was selected for quantitative surface roughness analysis using atomic force microscopy (AFM). The surface morphology was investigated using a Bruker Dimension Icon AFM system (Santa Barbara, CA, USA) operated in PeakForce Tapping mode (Bruker, Billerica, MA, USA) with silicon nitride probes featuring sharp tips (tip radius ~ 2 nm). A representative AFM image over a 1 × 1 μm^2^ area of the 44 nm TiO_x_ film is illustrated in Figure 2c. The surface roughness was quantified by calculating the root mean square (RMS) roughness from AFM height data collected over a 10 × 10 μm^2^ scan area. The RMS roughness was determined to be 0.2 ± 0.05 nm, indicating a homogeneous surface with small grains, consistent with observations from the SEM analysis.

**Figure 3:**
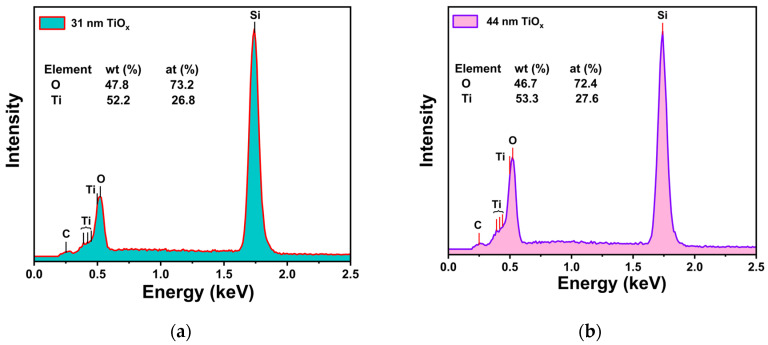
EDX spectrum of TiO_x_ thin films for two different thicknesses, (**a**) 31 nm and (**b**) 44 nm.

Figure 3 shows the EDX spectrum of the TiO_x_ films. The chemical compositions of the films were determined to be Ti_26.8_O_73.2_ for 31 nm and Ti_27.6_O_72.4_ for 44 nm TiO_x_, indicating a slight excess of oxygen content relative to the stoichiometric composition of TiO_2_ (Ti_33.3_O_66.7_).

**Figure 4:**
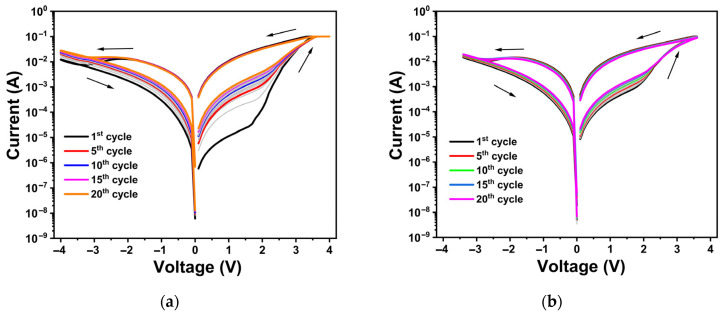
The semi-logarithmic I-V curves (20 cycles) of the Al/TiO_x_/TiN/Si(n^++^)/Al memristor with a TiO_x_ thickness of (**a**) 31 nm and (**b**) 44 nm. A current compliance of I_c_ = 100 mA was used during the measurements, and the arrow indicates the sweep direction. The light gray lines indicate in-between cycles.

To investigate the electrical behavior of TiO_x_ thin films, I–V measurements were conducted on asymmetric Al/TiO_x_/TiN/Si(n^++^)/Al structures. A voltage bias was applied exclusively to the top electrode (TE), while the bottom electrode (BE) was maintained at ground potential. Figure 4a,b illustrate the I–V curves for devices with 31 nm and 44 nm TiO_x_ thin films as the insulating layers, respectively. Both devices demonstrated forming-free switching behavior, characterized by a homogenous transition to LRS (SET) when a positive voltage was applied and a return to HRS (RESET) under a negative voltage. This gradual resistance change suggests the absence of conductive filament (CF) formation in the TiO_x_ layers. For the 31 nm and 44 nm films, the SET voltages were 3.5 V and 3.6 V, respectively. The RESET voltages were observed at approximately −3.4 V for the 31 nm film and −3.1 V for the 44 nm film.

During the RESET operation, the current began to decline from −2.0 V for the 31 nm film and from −1.8 V for the 44 nm film, continuing to drop until reaching the RESET voltage in both cases. This region, known as the negative differential resistance (NDR) region, is characterized by an increase in resistance as the voltage increases. An ideal NDR region is particularly interesting for oscillators and diodes [51]. The observed non-filamentary switching behavior is primarily linked to the modulation of the Schottky barrier at the metal–oxide interface [52].

A comprehensive understanding of the physical mechanisms driving resistive switching is essential for advancing memristive device optimization and commercial development. Despite decades of extensive research, the exact nature of resistive switching remains a topic of ongoing debate. The electrical conduction in the MIM structure may not be fully captured through simple I–V fitting using predefined models. However, it is often possible to identify the main current pathway within the device among the various possible conduction mechanisms. In general, a non-linear I–V curve can result from various charge transport mechanisms, such as Schottky or thermionic emission, Poole–Frenkel (P–F) emission, and space-charge-limited current (SCLC) conduction [53]. Specifically, the Schottky mechanism is typically characterized by a log(I) ∝ V^1/2^ dependence, while the SCLC mechanism follows a quadratic relationship, expressed as I ∝ V^2^ [54,55,56].

**Figure 5:**
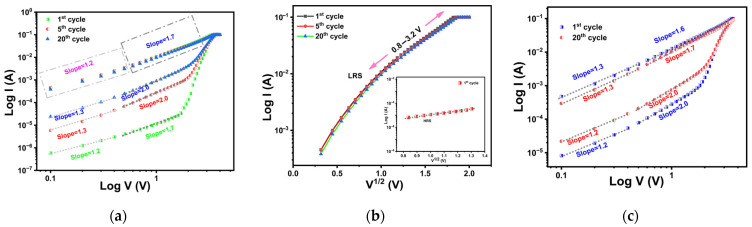
(**a**) Double-logarithmic I–V plots and (**b**) log I–V^1/2^ plots for the 1st, 5th, and 20th cycles_._ The inset shows the corresponding log I–V^1/2^ plot in HRS for the 1st cycle of 31 nm TiO_x_, under positive bias. (**c**) Double-logarithmic I–V plots for the 1st and 20th cycles of 44 nm TiO_x_ in the positive bias region.

Figure 5a displays the double-logarithmic I–V fitting for the 31 nm TiO_x_ film under positive voltage bias. During the first positive bias sweep, the slopes of the log I–log V plot of HRS changed from 1.2 to 1.7, followed by a sharp increase in current with increasing voltage. As the voltage sweep was repeated, the slopes in the HRS region increased further, reaching 1.3 at a lower bias and 2.0 at a higher bias. For LRS, the I–V curve showed a linear behavior with a slope of approximately 1.2 in the low-bias region, indicating ohmic conduction. At higher bias, the slope increased to 1.7, and the data fit well with a log I–V^1/2^ plot, as illustrated in Figure 5b. This suggests that the conduction in LRS is governed predominantly by ohmic and Schottky emission mechanisms. The inset of Figure 5b presents the fitting for the first HRS cycle at higher bias, which also fitted well to the log I–V^1/2^ plot. This observation indicates that, in HRS, the conduction initially transitions from ohmic in the low-field region to Schottky emission in the high-field region. With repeated cycling, the dominant mechanisms evolve towards a combination of ohmic and space-charge-limited conduction (SCLC).

Figure 5c shows the double-logarithmic I–V curves for the 44 nm TiO_x_ film under positive bias. Regardless of the number of cycles, HRS displayed a change in slope from 1.2 to 2.0, followed by a sharp increase in current as voltage increases, consistent with the trap-controlled SCLC model [57]. In LRS, the log I–log V curves showed a slope of approximately 1.3 at low bias and 1.7 at high bias, again suggesting that ohmic and Schottky emission mechanisms dominate the conduction process.

**Figure 6:**
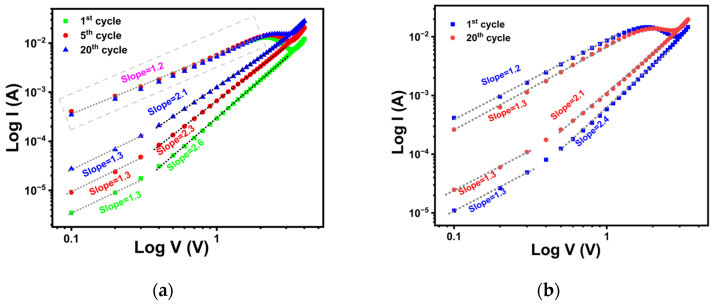
(**a**) Double-logarithmic I–V plots for the 1st, 5th, and 20th cycles of 31 nm TiO_x_, (**b**) double-logarithmic I–V plots for the 1st and 20th cycles of 44 nm TiO_x,_ in the negative bias region.

Figure 6a,b illustrate the double-logarithmic I–V curves of the 31 nm and 44 nm TiO_x_ films, respectively, under negative voltage bias. In HRS, the slope of the log I–log V plot for the 31 nm film changed from 1.3 to 2.6, while for the 44 nm film, it varied from 1.3 to 2.4 with increasing voltage. These results indicate ohmic conduction at a lower bias and SCLC at a higher bias in HRS for both films. With increasing cycle number, the slope of the ohmic region remained nearly constant at 1.3 for both resistance states. However, the slope of the SCLC region decreased from 2.6 to 2.1 for the 31 nm film and from 2.4 to 2.1 for the 44 nm film, respectively. Notably, the slope at higher bias in HRS decreases with successive cycles, suggesting electric field-induced charge trapping in the TiO_x_ film [58]. In contrast, the LRS of both films exhibited a linear relationship between log I and log V, indicating ohmic conduction across the bias range.

**Figure 7:**
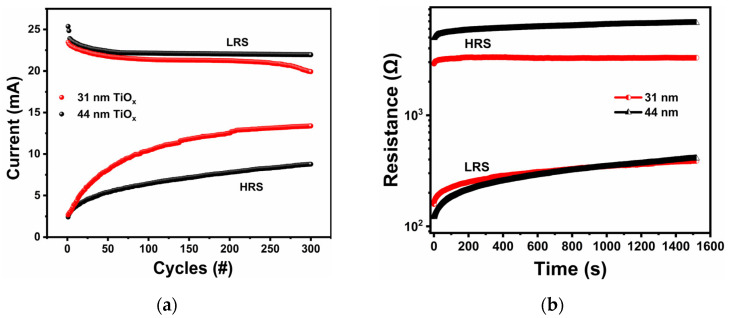
(**a**) Endurance plot for 31 and 44 nm TiO_x_ thin-film HRS and LRS currents read at 2.0 V (300 cycles, Ic = 100 mA). (**b**) Retention performance of the devices based on 31 and 44 nm TiO_x_ thin films, read at 0.5 V.

Figure 7a presents the endurance performance of the fabricated TiO_x_-based memristors, highlighting consistent and repeatable resistive switching behavior over 300 DC cycles. The HRS and LRS currents were recorded at a read voltage of 2.0 V. The variation of the current in LRS was insignificant, while a noticeable gradual increase in HRS current was observed with increasing resistive switching cycle number. Notably, these variations were more pronounced in the 31 nm TiO_x_ film than in the 44 nm film, indicating that the 44 nm film exhibits better stability and endurance in resistive switching performance. The observed increase in HRS current over repeated cycling suggests a reduction in trap density within the TiO_x_ layer, which enhances carrier transport across the Al/TiO_x_/TiN structure [59]. The fatigue in the RS behavior in the successive cycles can be explained by the variation of vacancy densities and oxygen ions [60]. The observed increase in HRS current with cycling is consistent with the recent literature findings [61]. It is attributed to interface degradation at the TiO_x_/TiN interface. Studies have identified mechanisms such as ion accumulation, interfacial roughening, and chemical instability as key contributors. In particular, repeated switching cycles lead to the accumulation of oxygen vacancies near the TiO_x_/TiN interface, which enhances trap-assisted tunneling and results in an increase in HRS current. Under prolonged cycling, partial reduction or nitridation of the TiO_x_ layer at the TiN interface may form a TiO_x_N_y_ phase, thereby modifying the local defects and barrier height [61,62,63]. The ability of memristors to retain data plays a crucial role in developing highly stable memory devices. The retention characteristics of the devices utilizing TiO_x_ films with thicknesses of 31 nm and 44 nm are shown in Figure 7b. The measurements were conducted under ambient conditions at room temperature. During the retention test, resistance states were monitored using a read voltage of 0.5 V. The results indicate strong non-volatile behavior in both HRS and LRS, with clear separation maintained between the two states. Although a minor decline in LRS was observed for both types of devices over the 1500 s measurement duration, the overall stability remained good throughout the test. The devices based on the 44 nm TiO_x_ layer demonstrated a greater ON/OFF ratio than those with the 31 nm layer. However, the results suggest that both film thicknesses exhibited relatively stable performance.

**Figure 8:**
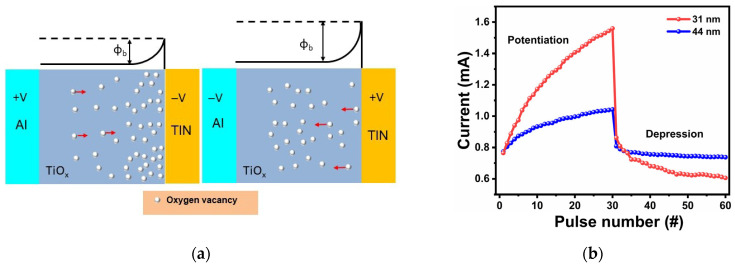
(**a**) Schematic illustration of the proposed model depicting the switching mechanism. (**b**) Potentiation and depression behavior of 31 nm and 44 nm thin-film-based memristor synapses under stimulation by 30 identical positive voltage pulses (+2 V, 200 ms) followed by 30 identical negative voltage pulses (−2 V, 200 ms).

This type of RS mechanism consists of an interface-type model resulting from Schottky barrier height modulation caused by charge trapping and de-trapping at the TiN/TiO_x_ interface under external electric fields, as presented in Figure 8a. The Al top electrode in contact with TiO_x_ forms an effective ohmic contact due to the close match between the work function of Al (~4.1 eV) and the electron affinity of TiO_x_ (~4.0 eV) [64,65]. However, TiN has a work function of ~4.7 eV [66], so it is reasonable to assume the occurrence of a Schottky barrier at the TiN/TiO_x_ interface. Thin films deposited at room temperature tend to exhibit a higher concentration of oxygen vacancies near the bottom interface, often attributed to the existence of reduced Ti^3+^ [43]. According to Park et al., the upper surface of spin-coated films becomes more stoichiometric because it is the only side exposed to atmospheric conditions during deposition, resulting in a gradient of oxygen vacancies across the film thickness [67]. The RS effect is associated with the movement of these oxygen vacancies under an electric field, which modulates the potential barrier at the TiN/TiO_x_ interface. These vacancies introduce extra interface potential, effectively lowering the Schottky barrier height [68]. When a positive bias is applied to the Al TE, the TiN BE is under negative bias, attracting oxygen vacancies (positive charge traps) toward the TiN/TiO_x_ interface. This leads to electron de-trapping and migration away from the interface, causing a shift in the local potential and Fermi level alignment, which reduces the Schottky barrier height at the BE interface. As a result, the electrons can move easily through the barrier and trigger a transition from HRS to LRS [69]. Conversely, applying a negative bias to the TE (BE is at a positive bias) causes the oxygen vacancies to migrate away from the TiN/TiO_x_ interface. In addition, oxygen vacancies situated in the vicinity of the BE interface can trap electrons during electron movement as well. As electrons are injected and begin to occupy these trap states, the local positive charge is partially neutralized, leading to an increase in the Schottky barrier height. This reduces the free carrier density and raises the interfacial resistance, thereby switching the device from LRS back to HRS [70]. This interface-type RS behavior, involving Schottky barrier modulation due to oxygen vacancy migration or electron trap dynamics, has been widely observed in TiO_x_ and other resistive switching materials [43,59,67,71,72]. Thus, the Al/TiO_x_/TiN device operates through interface-type RS, characterized by Schottky barrier height modulations at the TiN/TiO_x_ interface that can be attributed to the oxygen vacancy migration at the interface.

To replicate key functionalities of biological synapses using the Al/TiO_x_/TiN memristor, pulsed current–voltage (I–V) measurements were conducted for devices with both 31 nm and 44 nm TiO_x_ film thicknesses. In this context, the applied voltage pulses act as external stimuli, while the resulting current responses represent changes in synaptic weight. A critical feature of biological synapses is their ability to modulate synaptic strength incrementally in response to repeated stimuli, known as long-term potentiation (LTP) and long-term depression (LTD). These synaptic behaviors can be emulated in memristors by controlling the direction and duration of the applied voltage pulses, in line with the flux-controlled memristor model [73]. When 30 consecutive positive voltage pulses (2 V, 200 ms) are applied, the conductance of the memristors gradually increases. Conversely, applying 30 successive negative pulses (−2 V, 200 ms) leads to a gradual reduction in conductance as illustrated in Figure 8b. This bidirectional modulation corresponds to LTP (increased conductance) and LTD (decreased conductance), both of which are crucial for mimicking the adaptive learning processes observed in computational neuroscience, neural networks, and biological systems [74].

The analog and non-volatile switching characteristics of these devices make them highly suitable for implementation as artificial synapses in neuromorphic computing systems [75]. Their ability to achieve continuous resistance modulation enhances fault tolerance and enables high-density data storage. Future investigations should aim to address the remaining challenges, including temperature-dependent stability, long-term data retention, scaling effects on resistive switching behavior within crossbar array architectures, and a detailed evaluation of synaptic plasticity characteristics in Al/TiO_x_/TiN memristors.

## 4. Conclusions

In this study, TiO_x_-based Al/TiO_x_/TiN memristive devices were fabricated on a highly doped n-type Si substrate, exhibiting analog resistive switching behavior. Structural analysis confirmed that the sputter-deposited TiO_x_ films are initially amorphous when solely deposited on Si but crystallize into a mixed anatase–rutile phase when sandwiched between TiN and Al electrodes. These films are slightly overoxidized compared with stoichiometric TiO_2_. Surface morphology analysis showed that the 31 nm TiO_x_ film is relatively smooth, whereas the 44 nm film displays a slightly rougher texture due to a higher density of agglomerates. The analysis of the current-conduction mechanism revealed that in HRS, current conduction is mainly governed by ohmic behavior at low bias and SCLC at high bias. In LRS, ohmic conduction dominates at low bias, while Schottky emission prevails at high bias. The devices demonstrated excellent endurance, maintaining stable performance over more than 300 DC switching cycles. Furthermore, evaluation of the retention time in both HRS and LRS confirmed that the resistance states remained stable, with minimal degradation observed in LRS over 1500 s. The resistive switching mechanism is attributed to the drift of oxygen vacancies, which modulate the Schottky barrier at the TiN/TiO_x_ interface. Additionally, the memristors effectively exhibited strong LTP and LTD characteristics. Among the tested structures, the memristor with a 44 nm TiO_x_ film showed superior performance in terms of switching stability, ON/OFF ratio, and endurance. Overall, TiO_x_ film thickness plays a crucial role in optimizing device performance. The fabricated Al/TiO_x_/TiN memristive devices exhibit promising characteristics for future applications in non-volatile memory and neuromorphic computing devices.

## Data Availability

The original contributions presented in this study are included in the article. Further inquiries can be directed to the corresponding author.
